# Does Zoledronic Acid Provide a Good Clinical Outcome in Patients With Chronic Back Pain Associated With Vertebral Osteoporosis?

**DOI:** 10.7759/cureus.33328

**Published:** 2023-01-03

**Authors:** Umesh M, Prabhu Ethiraj, Siyad M Nazar, Sandesh Agarawal, Arun H Shanthappa

**Affiliations:** 1 Department of Orthopedics, Sri Devaraj Urs Medical College, Sri Devaraj Urs Academy of Higher Education and Research, Kolar, IND

**Keywords:** t-score, zoledronic acid, bisphosphonates, bone mineral density, dual energy x-ray absorptiometry, fragility fractures, bone remodeling, vertebral osteoporosis

## Abstract

Background

Osteoporosis is a chronic, progressive, systemic condition of the skeletal tissue that is characterized by reduced bone density, microarchitecture deterioration, and fragile bones, making osteoporotic fractures or fragility fractures more likely to occur. This condition often remains asymptomatic and undiagnosed until it presents with fragility fractures. The condition is associated with a significant socioeconomic burden with disability, morbidity, and mortality. Therefore, early diagnosis, as well as treatment, is needed to prevent fractures. Intravenous zoledronic is an effective bisphosphonate with high patient compliance due to once-yearly dosing. The present study aims to determine whether zoledronic acid effectively treats chronic back pain in people with osteoporosis.

Materials and methods

Seventy patients above the age of 60 years presented with complaints of chronic low back aches to the outpatient department of orthopedics, R L Jalappa Hospital & Research Centre attached to Sri Devaraj Urs Medical College. The study was conducted between November 2016 and November 2018.

Results

All the patients found excellent clinical improvement following zoledronic acid infusion in early and long-term follow-ups. Additionally, it was found that zoledronic acid's effectiveness was excellent, with significant improvement in bone mineral density (BMD), T-score, and Z-score.

Conclusion

Early diagnosis and treatment of vertebral osteoporosis is the most important factor in preventing fragility fractures. Zoledronic acid, an antiresorptive drug with better compliance, is very effective in controlling low back pain, improving bone mineral density, and preventing the occurrence of atraumatic fragility fractures. With all the above factors, zoledronic acid is a preferable bisphosphonate for the treatment and prevention of osteoporosis compared to other modalities of treatment of osteoporosis.

## Introduction

Osteoporosis is a chronic progressive systemic skeletal disease characterized by reduced bone mass and micro-architectural deterioration of bone tissue with consequent fragile bones, predisposing to increased risk of 'osteoporotic fractures' or 'fragility fractures [1,2]. This condition often remains asymptomatic and undiagnosed until it presents with fractures involving the hip, spine, proximal humerus, pelvis, and wrist resulting from low-velocity trauma, frequently leading to hospitalization [3]. Seldom it also presents with severe backache or loss of height. American Orthopaedic Association (AOA) Own the Bone program has given orthopedic surgeons a vital role in managing osteoporosis beyond acute fracture management, which has been proven useful in achieving better patient-related outcomes [4]. According to WHO's published statistics, one out of three women and one out of eight men in India over age 50 are osteoporotic. It is estimated in a few studies that more than 61 million Indians are osteoporotic, of which 80% are females [5,6]. The earliest clinical feature of osteoporosis is usually chronic low back ache which can be associated with a wide spectrum of diseases and is often underdiagnosed. Hence, many cases present in the health care system with osteoporotic fractures. The most common fractures seen in osteoporotic patients are vertebral compression fractures, followed by hip fractures [7]. Both anabolic and antiresorptive therapies are available to use separately or one after another and even in combination for osteoporosis [8]. Since the 1960s, after the discovery of bisphosphonates, the management of osteoporosis has revolutionized [9]. These are pyrophosphate-stable synthetic analogs suppressing osteoclasts' bone resorption and indirectly decreasing osteoblast activity. Teriparatide, an analog of parathyroid-hormone (PTH); abaloparatide, an analog of parathyroid hormone-related peptide (PTHrP); romosozumab, an investigational monoclonal antibody that inhibits sclerostin [10,11]; nitrogen-containing bisphosphonates; denosumab (a RANKL blocker); estrogen, and selective estrogen receptor modulators (SERMs), are the main antiresorptive drugs available at present. Denosumab is credited with the most rapidly acting and potent antiresorptive properties [12,13]. Intravenous zoledronic acid, when given as a 5 mg infusion over 15 minutes once yearly, is a potent and compliant bisphosphonate.

## Materials and methods

This prospective study was conducted on 70 patients above 60 years of age who presented with a chronic low back ache to the Outpatient Department of Orthopaedics, R L Jalappa Hospital and Research Centre attached to Sri Devaraj Urs Medical College after obtaining clearance from the institutional ethics committee (SDUAHER/KLR/PG-DIS/380/2019-20). The subjects for the study were selected fitting the inclusion and exclusion criteria as mentioned in Table 1.

**Table 1:** Inclusion and exclusion criteria. NSAIDs: Nonsteroidal anti-inflammatory drugs.

Inclusion Criteria	Exclusion Criteria
Patients of either sex aged above 60 years.	Patients with primary and/or secondary tumors of the spine.
Patients experiencing focal back pain which was insidious in onset for more than six weeks of duration.	Patients on bisphosphonates therapy.
Pain not relieved by NSAIDs , opiods and physiotherapy	Patients with traumatic fractures of the spine and radiculopathy.

They were selected for this study after taking informed consent. Demographic data, history, clinical examination, and details of investigations were recorded in the study proforma. In addition, the baseline visual analog score and Modified Oswestry low back pain and disability assessment scores were recorded. The assessment tools used are radiographs, dual-energy X-ray absorptiometry (DEXA scan), routine blood investigations, electrocardiograms for the selected patients, and lumbosacral spine anteroposterior and lateral view radiographs. In addition, the patients having osteoporotic features in radiographs were advised for DEXA 10 of spine anteroposterior assessment. In our study, bone density was assessed using GE Healthcare Prodigy encore-based DEXA scan. Those patients who were found to be osteoporotic as per WHO definition criteria were taken into this study.

After explaining the study and possible adverse events of a 5 mg infusion of zoledronic acid, consent was taken. Blood investigations and ECG were done to find contraindications to zoledronic acid infusion. The patients without contraindications were advised to take sufficient fluids orally for adequate hydration. Later 5 mg zoledronic acid infusion was given for a minimum duration of 15 minutes. The patients were monitored for any allergic reaction and other immediate adverse events for one day and were recorded. Prophylactic antipyretic medication paracetamol was given to all patients. Upon discharge, patients received advice on back strengthening exercises, and oral vitamin D and calcium supplements were given to all patients for a whole year. The analgesics were avoided to assess the exact effect of zoledronic acid. They were followed up and assessed for pain and functional ability improvement using a visual analog scale (VAS) and modified Oswestry low back pain disability index (MODI) (at 12 weeks, 24 weeks, and one-year follow-up). In the final follow-up after a year, every patient underwent bone density assessment by DEXA. Assessment protocols for the initial visit, follow-up, and final visits are mentioned in Tables 2-4, respectively.

**Table 2:** Assessment at initial visit. VAS: Visual analog scale; DEXA: Dual energy X-ray absorptiometry.

Initial or Baseline Assessment:
1 . Screening the patients clinically for factors suggestive of osteoporosis.
2. VAS chart and modified Oswestry back pain and disability questionnaire were given, and baseline scores were recorded.
3. Radiographs of the lumbar spine were taken to assess for osteoporosis.
4. Patients suspected of having osteoporosis were evaluated with a DEXA scan of the spine.
5. Patients who turned out to be osteoporotic on DEXA with a T-score of - 2.5 and below were evaluated with routine blood investigations and ECG to rule out contraindications for infusion.
6. Patients who did not have contraindications were admitted, and 5 mg zoledronic acid infusion was administered over a minimum duration of 15 min under monitoring and observed for one day for adverse events and allergic reactions.
7. Calcium 500 mg and vitamin D3 600000 IU supplement were advised.

**Table 3:** Assesment at second and third visits VAS: Visual analog scale; MODI: Modified Oswestry low back pain disability index.

Assessment at weeks 12 and 24
1. Clinical examination of patients.
2. Assessment of improvement in pain and function by recording VAS and MODI.
3. Advised to continue calcium and vitamin D3 supplement.

**Table 4:** Assessment at final visit. VAS: Visual analog scale; MODI: Modified Oswestry low back pain disability index; DEXA: Dual energy X-ray absorptiometry.

Assessment at 1 year:
1. Clinical examination of patients.
2. Assessment of final improvement in pain and function by recording VAS and MODI.
3. Assessment of final improvement in bone density using the DEXA scan.
4. Study completed.

## Results

The study includes 70 patients with female predominance, 45 female and 25 male patients. The average age of the patients in the study is 68.47 years, ranging from 61 years to 82 years. The gender-specific age distribution tells us that risk of osteoporosis in females is at an early age compared to males, as shown in Table 5.

**Table 5:** Gender-specific age distribution.

Age in years	Male	Female	Total
60-65 Years	1	15	16
65-70 Years	8	15	23
70-75 Years	10	14	24
>80 Years	1	-	1
Total	25	45	70

**Figure 1:**
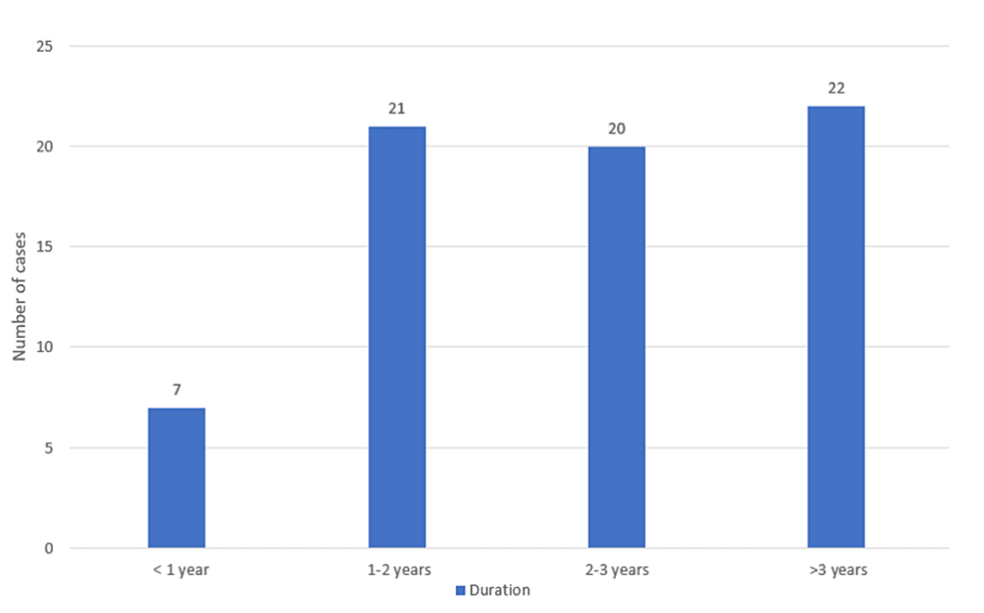
Duration of symptoms.

Patients in our study exhibited variable duration of symptoms ranging from 6 months up to 10 years, as depicted in Figure 1.

In the study we conducted, out of 70 patients, 21 had type II diabetes, eight had essential hypertension, and six had both. The remaining 35 patients had no comorbidities. The average baseline BMD done at the first visit was 0.798 gm/cm2 and ranged from 0.520 to 0.910 gm/cm2. The average final BMD done at 1-year follow-up visit was 0.946 gm/cm2, which ranged from 0.701 to 1.090 gm/cm2. The values are shown in Table 6.

**Table 6:** Patient distribution according to bone mineral density in first visit and final follow-up. BMD: Bone mineral density.

BMD in gm/cm^2^	No. of patients in the first visit	No. of patients at 1-year follow-up
0.500-0.600	1	0
0.601-0.700	4	0
0.701-0.800	22	4
0.801-0.900	34	14
0.901-1.000	2	29
1.001-1.100	0	16

**Figure 2:**
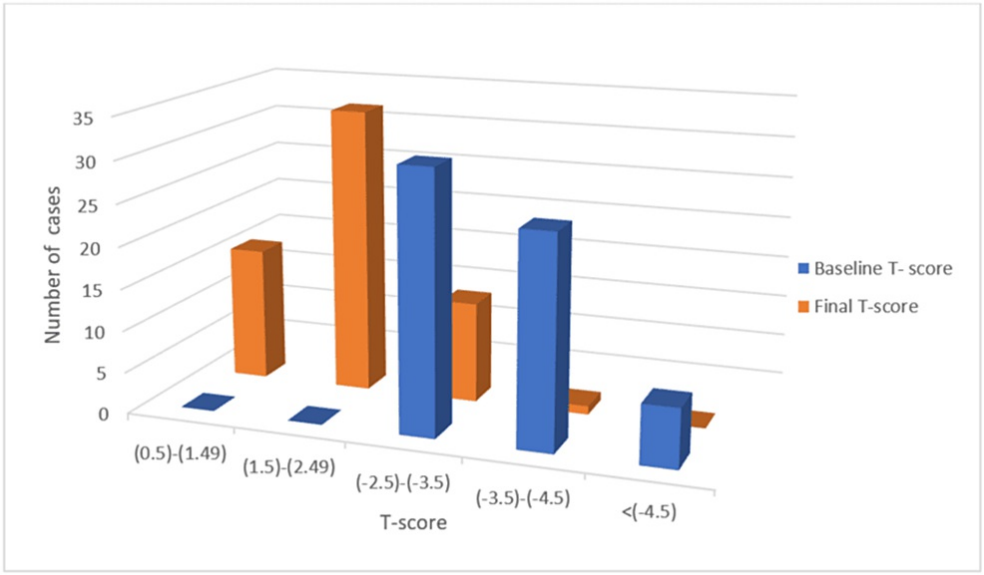
Patient distribution according to baseline and final T-score.

The average baseline T-score is -3.60, which ranges between -2.50 and -6.40 and the average final T-score is -1.90, which ranges between -0.70 and -3.92. The distribution of patients according to baseline and final T- score is shown in Figure 2. 

In our study, few adverse reactions developed only during the first week after infusion of zoledronic acid. Ten patients had no adverse effects. Headache was the most frequently observed adverse effect, seen in 42 patients the next day after infusion. Thirty-four patients had a fever in the evening or the next day after infusion. Fifteen patients complained of palpitation immediately after transfusion for about ½ to 1 hour. No patients had allergic reactions or arrhythmias. The same is shown in Table 7.

**Table 7:** Incidence of adverse events.

Adverse Events	No of patients
No. of adverse events	10
Headache	42
Fever	34
Post Transfusion Palpitation	15
Allergic Reactions	Nil
Arrhythmias	Nil

The average and trend of the VAS score and MODI score assessed at each follow-up are shown in Table 8.

**Table 8:** Average VAS and MODI scores. VAS: Visual analog score; MODI: Modified Oswestry low back ache disability index.

Duration	Change in VAS	MODI score
On presentation	5.95	47.55
12 weeks	4.16	31.84
24 weeks	9.68	29.2
1 year	2.54	18.98

Statistical assessment of the collected data was done using IBM SPSS Statistics, version 21 22.0.0.0. The calculated values are shown in Table 9.

**Table 9:** Statistical calculations by paired student t-test. BMD: Bone mineral density; VAS: Visual analog score; OPI: Oswestry pain index; FU: Follow-up; DF: Degree of freedom.

Pairs	Paired Differences					T-value	DF	P-value
	Mean	Std Deviation	Std Error Mean	95% Confidence Interval of Differences				
				Lower	Upper			
Baseline BMD-BMD at 1 year	0.14719	0.08847	0.011146	0.169471	0.12491	13.205	62	<0.0001
T-Score-T-Score at 1 year	1.37667	1.56736	0.19747	1.77140	0.98193	6.972	62	<0.0001
Z-Score-Z-Score at 1 year	1.37317	0.82730	0.10423	1.58153	1.16482	13.174	62	<0.0001
Baseline VAS-Baseline VAS at 1^st^ FU	1.7937	1.1095	0.1398	1.5142	2.0731	12.832	62	<0.0001
Baseline VAS-Baseline VAS at 2^nd^ FU	2.2698	1.4724	0.1855	1.8990	2.6407	12.236	62	<0.0001
Baseline VAS-Baseline VAS at 3^rd^ FU	3.4127	1.6813	0.2118	2.9893	3.8361	16.111	62	<0.0001
Baseline OPI-Baseline OPI at 1^st^ FU	15.7143	11.5834	1.4594	12.7970	18.6315	10.768	62	<0.0001
Baseline OPI-Baseline OPI at 2^nd^ FU	18.3492	12.4928	1.5739	15.2029	21.4955	11.658	62	<0.0001
Baseline OPI-Baseline OPI at 3^rd^ FU	28.5714	15.0287	1.8934	24.7865	32.3564	15.090	62	<0.0001

## Discussion

The current study consists of 70 patients of either sex, 25 males and 45 females, with lower back pain for more than six weeks duration and vertebral osteoporosis with few non-traumatic compression fractures of the spine. Out of a total of 70 patients, seven patients were lost in follow-up. Hence 63 patients who completed one year of follow-up and underwent repeat BMD assessment at the end of one year were considered for statistical calculation and assessment. In the study, the effect of zoledronic acid in decreasing pain is proven to be excellent when assessed improvement using VAS scoring with p-value <0.001 in early (3, 6 months) and long-term (1 year) follow-up. The study also showed excellent results in functional improvement when reviewed using the MODI questionnaire scoring with a p-value of <0.001 in early (3, 6 months) and long-term (1 year) follow-ups. When assessed by statistically calculating mean, the standard error means, SD, 95% CI of differences, and T-value by paired student t-test considering the degree of freedom as 62 (n-2). The p-value was found to be less than 0.0001, which meant the study result was statistically significant with excellent results. The most frequent adverse events identified in the study were headache, followed by fever, then post-transfusion palpitations.

No patients had allergic reactions, arrhythmic episodes, or jaw osteonecrosis. Considering all these observations, all the patients found excellent clinical improvement following zoledronic acid infusion in early and long-term follow-ups. No patients in the study developed new vertebral compression fractures (traumatic or atraumatic). This signifies that pain relief after zoledronic acid infusion may even be a result of the prevention of new compression fractures and strengthening of trabeculae of the spine along with its analgesic effect.

A study by Koivisto K et al. on the efficacy of zoledronic acid for chronic back pain showed that improvement in the intensity of chronic lower back ache (LBA) was more significant with zoledronic acid compared to placebo. They have also recommended it as an interesting treatment alternative for LBA with osteoporosis, which is challenging to treat with a conservative approach [14].

Orwoll E et al., in the study comparing IV zoledronate and oral alendronate, compliance with zoledronic acid is significantly better than with alendronate. The study also demonstrates that zoledronate is effective in treating osteoporosis in males [15].

Cauley J et al., in their research study on zoledronic acid, stated that treatment with zoledronic acid significantly reduced hospital admission duration and limited activity. Additionally, the study concluded that a three-year treatment with zoledronic acid significantly reduced disability and fracture compared with a placebo in women with osteoporosis [16].

Ramalingaiah A et al., in their study, stated that once a year, the zoledronic acid infusion has excellent compliance with minimal incidence of adverse effects. It has also been shown to improve pain during the first six months after infusion and modest improvement in BMD. On comparing with the results of our study, it has demonstrated excellent results in pain control and BMD improvement [17].

The study's limitations were that it was based on a single center, and a more extensive study population is needed for a much more accurate statistical analysis of the efficacy of zoledronic acid. Other lifestyle factors influencing osteoporosis, like involvement in physical activity, drinking and smoking habits, and dietary factors, were not considered. The study takes lower back aches as the chief complaint, which arises due to a spectrum of diseases. Also, the study was not a randomized controlled study. Furthermore, it did not compare the efficacy of oral vs. IV infusions of bisphosphonates.

## Conclusions

Chronic low back aches in elderly patients without any identifiable causes will usually be due to vertebral osteoporosis. In addition to affecting the individual's quality of life, vertebral osteoporosis can lead to substantial healthcare costs. Most of the population in this age group is often unaware of the consequences of age-related osteoporosis. Early diagnosis, appropriate treatment, and regular follow-up, when practiced among treating doctors, have proven to be essential factors in preventing fragility fractures and reducing lower back pain. Zoledronic acid, an antiresorptive drug with potent action and better patient compliance, is very effective in controlling low back pain, improving BMD, and preventing the occurrence of atraumatic compression fractures. With all the above factors, zoledronic acid can be considered a preferable bisphosphonate for the treatment and prevention of osteoporosis, with no known history of anaphylaxis or cardiac and renal impairment.
